# Comparison of the safety profile, conversion rate and hospitalization duration between early and delayed laparoscopic cholecystectomy for acute cholecystitis: a systematic review and meta-analysis

**DOI:** 10.3389/fmed.2023.1185482

**Published:** 2023-12-11

**Authors:** Hongsheng Wu, Biling Liao, Tiansheng Cao, Tengfei Ji, Jianbin Huang, Yumei Luo, Keqiang Ma

**Affiliations:** Department of Hepatobiliary Pancreatic Surgery, Affiliated Huadu Hospital, Southern Medical University, Guangzhou, China

**Keywords:** ELC, DLC, acute cholecystitis, review, meta-analysis

## Abstract

**Background:**

Although the past decade has witnessed unprecedented medical progress, no consensus has been reached on the optimal approach for patients with acute cholecystitis. Herein, we conducted a systematic review and meta-analysis to assess the differences in patient outcomes between Early Laparoscopic Cholecystectomy (ELC) and Delayed Laparoscopic Cholecystectomy (DLC) in the treatment of acute cholecystitis. Our protocol was registered in the PROSPERO database (registration number: CRD42023389238).

**Objectives:**

We sought to investigate the differences in efficacy, safety, and potential benefits between ELC and DLC in acute cholecystitis patients by conducting a systematic review and meta-analysis.

**Methods:**

The online databases PubMed, Springer, and the Cochrane Library were searched for randomized controlled trials (RCTs) and retrospective studies published between Jan 1, 1999 and Jan 1, 2022.

**Results:**

21 RCTs and 13 retrospective studies with a total of 7,601 cases were included in this research. After a fixed-effects model was applied, the pooled analysis showed that DLC was associated with a significantly high conversion rate (OR: 0.6247; 95%CI: 0.5115–0.7630; z = −4.61, *p* < 0.0001) and incidence of postoperative complications (OR: 0.7548; 95%CI: 0.6197–0.9192; z = −2.80, *p* = 0.0051). However, after applying a random-effects model, ELC was associated with significantly shorter total hospitalization duration than DLC (MD: −4.0657; 95%CI: −5.0747 to −3.0566; z = −7.90, *p* < 0.0001).

**Conclusion:**

ELC represents a safe and feasible approach for acute cholecystitis patients since it shortens hospitalization duration and decreases the incidence of postoperative complications of laparoscopic cholecystectomy.

**Systematic review registration:**

https://www.crd.york.ac.uk/PROSPERO/display_record.php?RecordID=389238, identifier (CRD42023389238).

## 1 Introduction

Laparoscopic Cholecystectomy (LC) represents the standard of care treatment for patients requiring a cholecystectomy (1, 2). Acute cholecystitis has long been considered unsuitable for immediate surgical treatment. However, with the significant progress achieved in minimally invasive technology, the number of patients undergoing early laparoscopic surgery has significantly escalated (3, 4). Nevertheless, no consensus has been reached on the optimal timing for surgery. It has long been thought that the risk of intraoperative conversion to laparotomy and intraoperative or postoperative complications was increased with early laparoscopic cholecystectomy (ELC) due to gallbladder congestion and edema, severe peripheral inflammatory reaction, and undefined anatomy of the Calot triangle (5–8). However, in recent years, with an improved knowledge of the etiology of the abovementioned complications and surgical method improvements, the incidence of intraoperative and postoperative complications has markedly decreased (9, 10). Compared to delayed laparoscopic cholecystectomy (DLC), ELC for acute cholecystitis reportedly decreases the operative complications and conversion rate and shortens the hospitalization duration (11–13). Nonetheless, the optimal timing for surgery remains subject to debate, emphasizing the need for further research.

Even though the superiority of ELC over DLC has been increasingly documented in the literature, most studies were based on relatively small populations (14–17). Accordingly, we comprehensively studied the current literature to determine the efficiency, safety profile, and potential benefits of ELC in contrast to DLC.

## 2 Methods

### 2.1 Data extraction

Literature was included in strict compliance with the PICOS principle. The target population consisted of “patients with acute cholecystitis,” the intervention was “LC,” the comparison was conducted between “ELC” and “DLC,” the outcomes consisted of “primary (conversion rate, intraoperative and postoperative complications) and secondary outcomes (Operation time, postoperative hospitalization duration and total hospitalization duration)” and the study design included “RCTs and retrospective studies.” The reporting principle of this meta-analysis complied with the Preferred Reporting Items for Systematic Review and meta-analysis (PRISMA) 2020 protocol and Meta-analysis of Observational Studies in Epidemiology (MOOSE) declaration (18, 19).

### 2.2 Method of literature-search

Our literature search was carried out in July of 2022 with no restriction to countries, type of publication or language utilized in the following electronic databases: PubMed and the Cochrane Library. The following MeSH terms and combinations were utilized to search the title, abstract and keyword sections: *Early Laparoscopic Cholecystectomy OR Delay Laparoscopic Cholecystectomy AND acute cholecystitis AND complication AND timing*.

### 2.3 Inclusion and exclusion criteria

All reports included in our study were randomized controlled trials (RCTs) or retrospective comparative studies (cohort or case–control studies) contrasted ELC to DLC irrespective of age, and analyzed at least one of our primary outcomes. During literature screening, the following were excluded: Animal experimental studies, case reports, letters to the editor and review articles.

### 2.4 Data extraction and outcomes of interest

The primary outcomes included intraoperative complication rate, postoperative complication rate and conversion rate. Intraoperative complications were common complications encountered during surgery, such as intraoperative bleeding, bile duct injury, and gallbladder perforation. In contrast, postoperative complications were defined as bleeding, wound infection, bile leakage, and so on (20, 21). Conversion was defined as open cholecystectomy performed when the anatomical structure around the gallbladder was unclear, pericholecystic inflammation was severe or intraoperative bleeding could not be controlled. The secondary outcomes were operative time (min), postoperative hospitalization duration (d) and total hospitalization duration (d). Comparative indicators included at least one primary outcome and one secondary outcome.

### 2.5 Quality assessment and statistical methods

The Cochrane Collaboration’s tool was used for assessing the risk of bias of RCTs graded as “low,” “unclear” or “high risk” (22). The Newcastle-Ottawa Scale (NOS) was employed for the quality assessment of retrospective research based on criteria categorized into three dimensions: selection, comparability, and outcomes. A total score > 5 was associated with a low risk of bias (23).

R software Version 4.1.3 was used to perform the meta-analysis of included studies. For continuous data, “metacont” from “meta” package was used to pool the data, and “metabin” was used for binary data. The pooled results of continuous and binary data were compared using weighted mean difference (WMD) and Odds Ratio (OR), respectively. When *I*^2^ < 50%, we used a fixed-effects model for pooling WMD or OR and its 95% confidence interval. Otherwise, a random-effects model was selected. Heterogeneity among the studies was explored by using subgroup analysis and meta-regression. To assess the pooled results’ stability, the “metainf” function was utilized for sensitivity analysis. Finally, Egger’s test and funnel plots were used to indicate the publication bias. When Egger’s *value of p*<0.05, the trim-and-fill approach was used for funnel plot asymmetry adjustment.

## 3 Results

### 3.1 Literature selection

**Figure 1:**
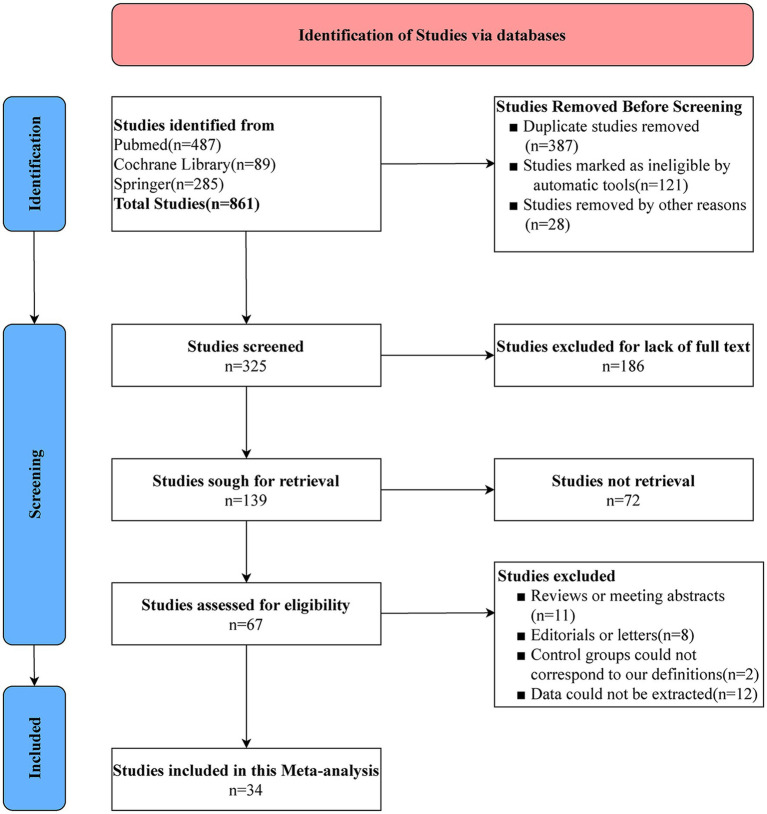
PRISMA flowchart of literatures screening.

861 studies were retrieved for a preliminary search in online databases PubMed, Springer, and Cochrane Library, according to the PRISMA2020 statement. After 827 non-eligible studies were excluded, ultimately, 34 studies were analyzed in this meta-analysis. The literature screening process is shown in Figure 1.

### 3.2 Quality assessment of the included studies

**Figure 2:**
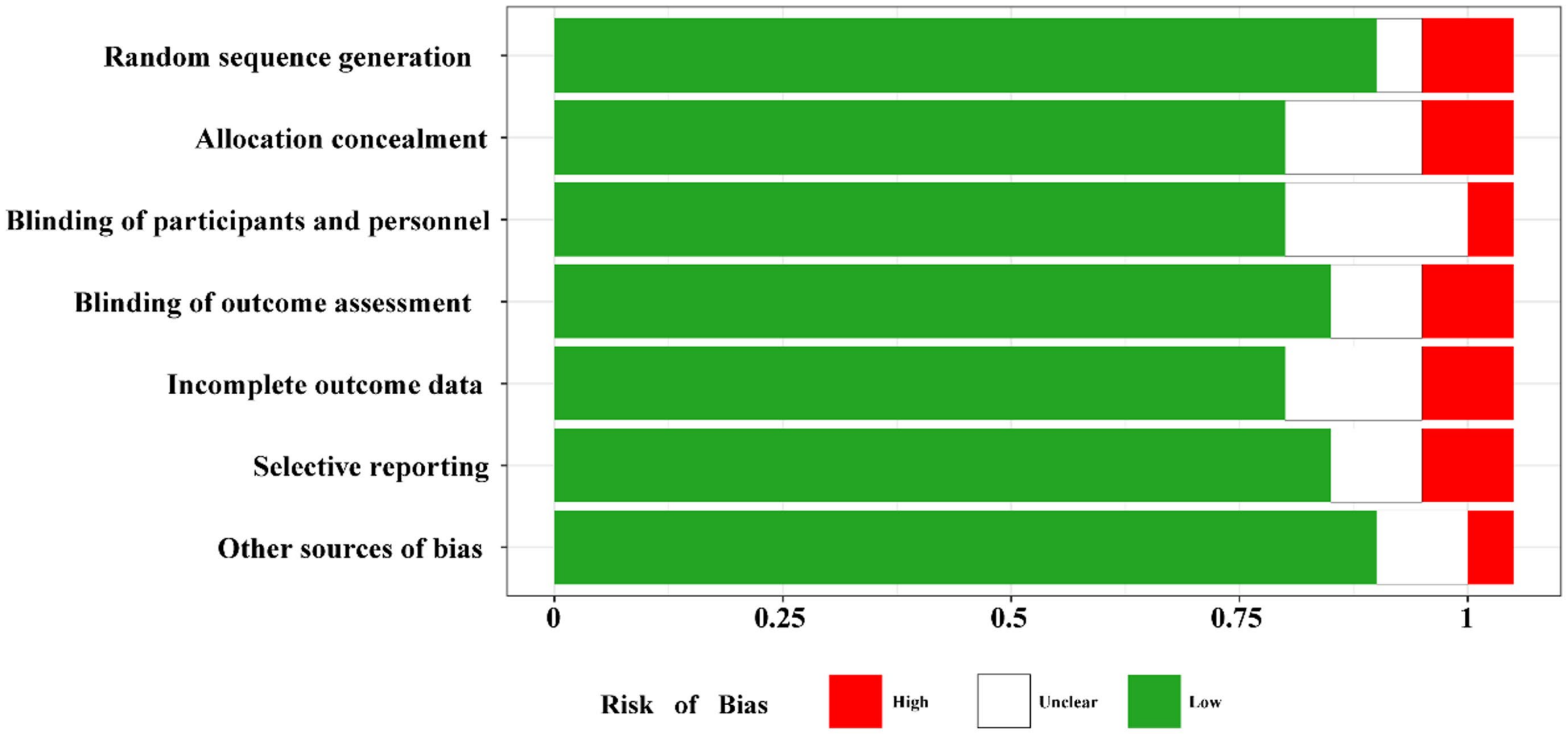
Cochrane bias risk assessment of including RCT studies.

The risk of bias in the included retrospective studies was evaluated using the Newcastle-Ottawa Scale based on the following domains subject selection, comparability and outcomes. The total scoring of each retrospective study was more than 5 points (Table 1). The Cochrane bias risk assessment system was employed to assess the included RCTs (Figure 2). Overall, the literature quality assessment found a low risk of bias for all included studies.

**Table 1:** Quality assessment of retrospective research base on Newcastle-Ottawa Scale.

Author(Year)	Selection of study population				Comparability	Outcome			Total Scoring
	Representativeness of the exposure cohort	Selection of non-exposed cohort	Ascertainment of exposure to implants	Demonstration that outcome of interest was not present at the start of study		Assessment of outcome	Was follow up long enough for outcomes to occur	Adequacy of follow up of cohorts	
Garber, SM 1997 (4)	★	★	★	★	★	★	★		7
Madan,AK 2002 (24)	★	★		★		★	★	★	6
Stevens, KA 2006 (25)	★		★	★	★	★		★	6
González-Rodríguez,F.J 2009 (26)	★	★		★	★	★	★	★	7
Chang, TC 2009 (27)	★	★	★		★		★	★	6
Choi, SB 2011 (28)	★	★	★	★	★	★	★	★	8
Falor, AE 2012 (29)	★	★	★	★	★	★	★		7
Zhu, Bin 2012 (30)	★	★	★	★	★		★	★	7
Panagiotopoulou 2012 (13)		★		★	★	★		★	5
Han, IW 2012 (15)	★		★	★	★		★		5
Kwon, YJ 2013 (31)	★	★		★	★		★	★	6
Gomes,RM 2013 (32)	★	★	★	★	★		★	★	7
Wu Hongsheng 2021 (33)	★	★		★	★	★		★	6

### 3.3 Characteristics of included studies

After literature screening and quality assessment, 34 studies were included in this meta-analysis, including RCTs (*n* = 21) (12, 14, 34–52) and retrospective studies (*n* = 13) (4, 13, 15, 24–33). The definition of ELC and DLC in the included studies was different. For ELC cases, laparoscopic cholecystectomy timing after acute cholecystitis onset was less than 24 h (n = 6) (12, 25, 35, 40, 46, 48), 48 h (*n* = 3) (24, 29, 51), 72 h (*n* = 15) (15, 26, 30–33, 36, 39, 42, 43, 45, 47, 49, 50, 52), 4 days (*n* = 3) (4, 37, 41), 5 days (*n* = 1) (34), and 7 days (*n* = 3) (13, 28, 38). For the 3 remaining studies (14, 27, 44), the specific timing for ELC was not mentioned, and the authors just proposed performing laparoscopic cholecystectomy as early as possible. Regarding the definition of DLC, laparoscopic cholecystectomy timing after acute cholecystitis onset was set to more than 24 h (n = 1) (25), 48 h (*n* = 2) (24, 29), 72 h (n = 7) (15, 26, 30–32, 39, 42), 6 weeks (*n* = 14) (12, 27, 35, 36, 38, 40, 41, 44, 45, 47–50, 52), 12 weeks (*n* = 1) (43) and 15 weeks (n = 1) (34). In 7 studies (4, 13, 14, 28, 33, 46, 51), the timing ranged from 72 h to 6 weeks, while no mention of the specific timing for DLC was found in 1 study (37). The details of the studies included in our report are shown in Table 2.

**Table 2:** The characteristics of including studies.

Author	Year	Country	Study design	Patients’NO.		Pathological characteristics	Definition of ELC	Definition of DLC	Observed outcomes
				**ELC**	**DLC**				
Lo, CM (34)	1996	China	RCT	27	25	NA	<5 days	>15 weeks	iii, v, vi
Garber, SM (4)	1997	USA	Retrospective	109	85	suppurative gangrenous	<4 days	>4 days	i, iii, iv, vi
Lai, PB (35)	1998	China	RCT	53	52	gangrenous	<24 h	6–8 weeks	i, iii, iv, v, vi
Lo, CM (36)	1998	China	RCT	45	41	NA	<72 h	8–12 weeks	i, iii, iv, v, vi
Chandler,*CF* (14)	2000	USA	RCT	21	22	suppurative	No mention	>5 days	i, ii, iii, iv, vi
Bhattacharya (37)	2002	UK	RCT	33	17	NA	<4 days	No mention	iii, iv, v, vi
Madan, AK (24)	2002	USA	Retrospective	14	31	NA	<48 h	>48 h	i, iii, iv, v, vi
Johansson,M (38)	2003	Sweden	RCT	74	69	NA	<7 days	6–8 weeks	i, iii, iv, vi
Serralta, AS (39)	2003	Spain	RCT	82	87	phlegmonous gangrenous	<72 h	>72 h	i, iii, iv, v, vi
Kolla, SB (40)	2004	India	RCT	20	20	NA	<24 h	6–12 weeks	i, ii, iii, iv, v, vi
Akyürek N (41)	2005	Turkey	RCT	31	30	NA	<4 days	8 weeks	i, v, vi
Stevens, KA (25)	2006	USA	Retrospective	132	121	suppurative	<24 h	>24 h	i, iii, iv, vi
Al-Mulhim (42)	2008	Saudi Arabia	RCT	82	114	NA	<72 h	>72 h	i, iii, iv, v, vi
González-Rodríguez,FJ (26)	2009	Spain	Retrospective	102	434	empyema suppurative	<72 h	>72 h	i, iii, iv, v, vi
Macafee,DA (43)	2009	UK	RCT	36	36	NA	<72 h	>12 weeks	i, ii, iii, v, vi
Yadav, RP (44)	2009	Nepal	RCT	21	22	NA	No mention	6–8 weeks	i, ii, iii, iv, vi
Chang, TC (27)	2009	China	Retrospective	56	33	suppurative	No mention	>6 weeks	iii, iv, v, vi
Choi, SB (28)	2011	Korea	Retrospective	57	59	NA	< 7 days	>7 days	iii, iv, v, vi
Falor, AE (29)	2012	USA	Retrospective	117	186	NA	<48 h	>48 h	i, iii, vi
Zhu, Bin (30)	2012	China	Retrospective	34	99	simple phlegmonous gangrenous	<72 h	>72 h	iii, iv, v, vi
Panagiotopoulou (13)	2012	UK	Retrospective	21	15	NA	< 7 days	>7 days	iii, vi
Han, IW (15)	2012	Korea	Retrospective	21	46	simple suppurative	<72 h	>72 h	1, ii, iii, iv, v, vi
Gul, R (45)	2013	India	RCT	30	30	NA	<72 h	6–8 weeks	i, iii, iv, vi
Gutt, CN (46)	2013	Germany	RCT	304	314	NA	<24 h	>7 days	i, ii, iii, vi
Kwon, YJ (31)	2013	Korea	Retrospective	33	28	empyema	<72 h	>72 h	i, iii, iv, v, vi
Gomes, RM (32)	2013	India	Retrospective	21	40	simple phlegmonous gangrenous	<72 h	>72 h	iv, vi
Ozkardeş,AB (47)	2014	Turkey	RCT	30	30	NA	<72 h	6–8 weeks	1, ii, iii, iv, vi
Agrawal, R (48)	2015	India	RCT	25	25	suppurative	<24 h	6–8 weeks	i, ii, iii, iv, v, vi
Rajcok, M (49)	2016	Slovakia	RCT	31	31	NA	<72 h	6–8 weeks	i, iii, iv, vi
Roulin, D (50)	2016	Switzerland	RCT	42	44	NA	<72 h	>6 weeks	i, ii, iii, iv, vi
Khalid (12)	2017	Pakistan	RCT	90	90	phlegmonous gangrenous	<24 h	6-12 weeks	i, ii, iii, iv, v
Davoodabadi (51)	2020	Iran	RCT	104	104	NA	<48 h	>7 days	i, iii, iv, vi
Isil, RG (52)	2021	Turkey	RCT	88	88	NA	<72 h	4–8 weeks	i, iii, iv, vi
Wu,Hongsheng (33)	2021	China	Retrospective	3,085	62	NA	<72 h	>7 days	i, iii, iv, v

ELC, Early Laparoscopic Cholecystectomy; DLC, Delay Laparoscopic Cholecystectomy; RCT, Randomized Controlled Trial; i, Conversion Rate; ii, Intraoperative Complications; iii, Postoperative Complications; iv, Operation Time; v, Postoperative Hospital Stay Time; vi, Total Hospital Stay Time.

### 3.4 Primary outcomes synthesis

**Figure 3:**
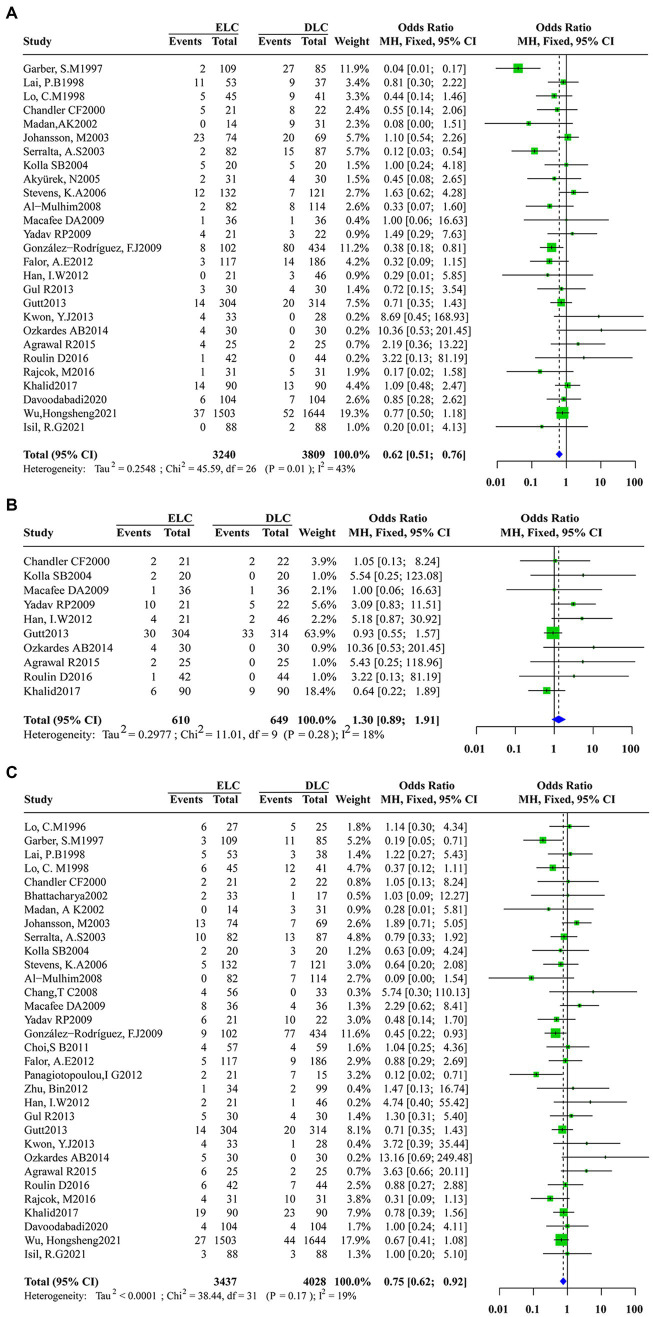
Forest plot of primary outcomes between ELC and DLC. **(A)**.Conversion rate; **(B)**.Intraoperative complications; **(C)**.Postoperative complications. Green squares represent the point estimates of the treatment effect OR, with 95% CI indicated by horizontal bars. Blue diamonds represent the summary estimate from the pooled studies with 95% CI using common fixed models.

I^2^ values less than 50% were obtained for the pooling conversion rate (I^2^ = 43%, τ2 = 0.2548, *p* = 0.01), intraoperative complications (*I*^2^ = 18.0%, τ2 = 0.2977, *p* = 0.28) and postoperative complications (*I*^2^ = 19.0%, τ2 < 0.0001, *p* = 0.17). Accordingly, we utilized a fixed-effects model to conduct the meta-analysis of primary outcomes. No significant differences in intraoperative complications were found between ELC and DLC (OR: 1.2616; 95%CI: 0.8998–1.7689; z = 1.35, *p* = 0.1778) (Figure 3B). However, compared with ELC, DLC associated with a high conversion rate (OR: 0.6247; 95%CI: 0.5115–0.7630; z = −4.61, *p* < 0.0001) (Figure 3A) and postoperative complication incidence (OR: 0.7548; 95%CI: 0.6197–0.9192; z = −2.80, *p* = 0.0051) (Figure 3C).

### 3.5 Secondary outcomes synthesis

**Figure 4:**
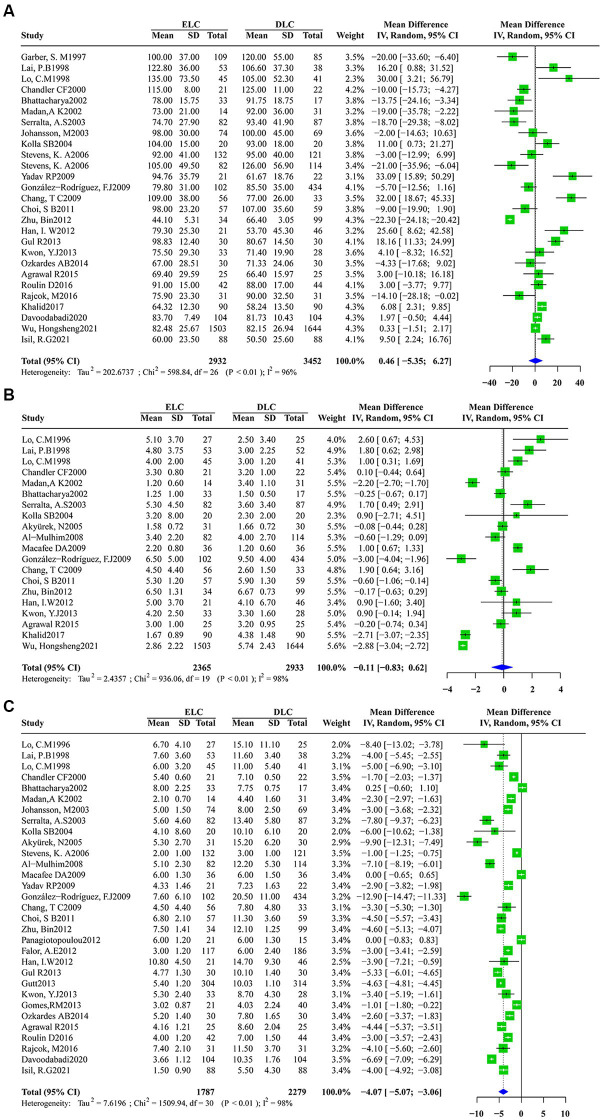
Forest plots of secondary outcomes between ELC and DLC. **(A)** Operation time; **(B)** Postoperative hospital stay duration; **(C)** Total hospital stay duration. Green squares represent the point estimates of the treatment effect OR, with 95% CI indicated by horizontal bars. Blue diamonds represent the summary estimate from the pooled studies with 95% CI using random effected models.

Significant heterogeneity was found among studies that assessed the secondary outcomes with I^2^ values above 50% obtained for the operation time (I^2^ = 96%, τ2 = 202.6737, *p* < 0.01), postoperative hospitalization duration (I^2^ = 98%, τ2 = 2.4357, p < 0.01) and total hospitalization duration (I^2^ = 98%,τ2 = 7.6196, p < 0.01). Accordingly, a random-effects model was applied for data synthesis. The pooled estimates revealed no marked differences in operation time (MD: 0.4594; 95%CI: −5.3527 to 6.2716; z = 0.16, *p* = 0.8769) (Figure 4A) and postoperative hospitalization duration (MD: -0.1088; 95%CI: −8.332 to 0.6157; z = −0.29, *p* = 0.7685) (Figure 4B) between ELC and DLC. In contrast, ELC was associated with significantly shorter total hospitalization duration than with DLC (MD: -4.0657; 95%CI:-5.0747 to −3.0566; z = −7.90, *p* < 0.0001) (Figure 4C).

### 3.6 Subgroup analysis for exploration of sources of heterogeneity

**Figure 5:**
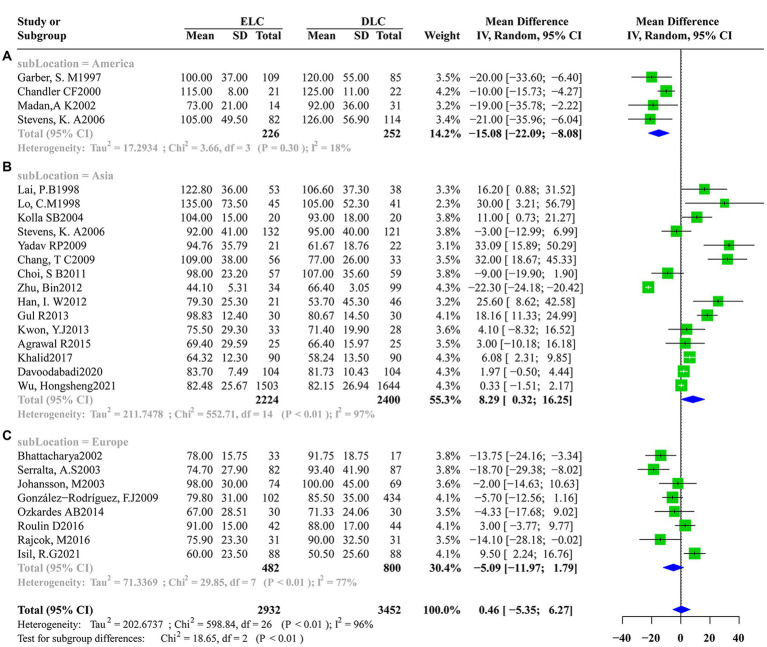
Subgroup analysis of location factors for operation time between ELC and DLC. **(A)** Subgroup for American population; **(B)** Subgroup for Asian population; **(C)** Subgroup for European population.

**Figure 6:**
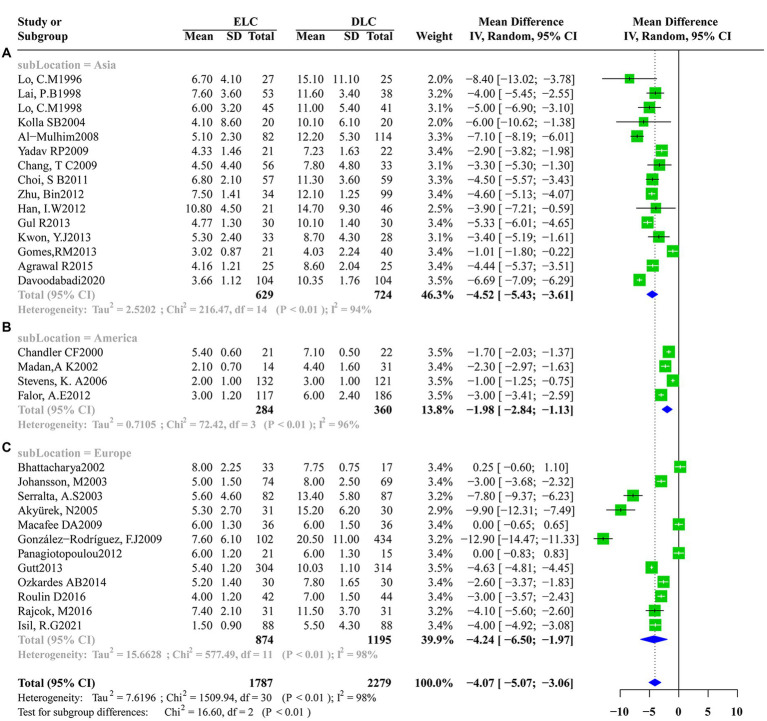
Subgroup analysis of location factors for total hospital stay duration between ELC and DLC. **(A)** Subgroup for Asian population; **(B)** Subgroup for American population; **(C)** Subgroup for European population.

Given that significant heterogeneity was found in operation time, postoperative hospitalization duration and total hospitalization duration, subgroup analysis was conducted according to the study design (e.g., RCT or Retrospective research), location (e.g., Asia, America or Europe), ELC definition (e.g., Timing of laparoscopic cholecystectomy after the onset of acute cholecystitis less than 72 h or other definitions) and year of study (e.g., Studies before 2013 or since and after 2013). Subgroup analysis according to operation time indicated no significant significance between the subgroups in the study design (heterogeneity test: *I*^2^ = 96%,τ^2^ = 202.674, *p* < 0.01, random effect model: *χ^2^* = 0.97, *df* = 1, *p* = 0.33) (Appendix 1), ELC definition (heterogeneity test: *I*^2^ = 96%,τ^2^ = 202.674, *p* < 0.01, random effect model: *χ^2^* = 0.01, *df* = 1, *p* = 0.92) (Appendix 2) and year of study (heterogeneity test: *I*^2^ = 96%,τ^2^ = 202.674, *p* < 0.01, random effect model: *χ^2^* = 0.80, *df* = 1, *p* = 0.37) (Appendix 3). However, after subgroup analysis according to location, compared with America and Europe, ELC was associated with longer operation time than DLC in Asia (heterogeneity test: *I*^2^ = 96%,τ^2^ = 202.674, *p* < 0.01, random effect model: *χ^2^* = 18.65, *df* = 2, *p* < 0.01) (Figure 5). Subgroup analysis according to the total hospitalization duration revealed no significant differences between subgroups for the study design (heterogeneity test: *I*^2^ = 98%,τ^2^ = 7.6196, *p* < 0.01, random effect model: *χ^2^* = 0.38, *df* = 1, *p* = 0.54) (Appendix 4), ELC definition (heterogeneity test: *I*^2^ = 98%,τ^2^ = 7.6196, *p* < 0.01, random effect model: *χ^2^* = 0.96, *df* = 1, *p* = 0.33) (Appendix 5) and year of study (heterogeneity test: *I*^2^ = 98%,τ^2^ = 7.6196, *p* < 0.01, random effect model: *χ^2^* = 0.07, *df* = 1, *p* = 0.79) (Appendix 6). However, ELC was associated with longer total hospitalization duration than DLC in Asia compared with America and Europe (heterogeneity test: *I*^2^ = 98%,τ^2^ = 7.6196, *p* < 0.01, random effect model: *χ^2^* = 16.60, *df* = 2, *p* < 0.01) (Figure 6).

### 3.7 Meta-regression for investigating sources of heterogeneity

**Figure 7:**
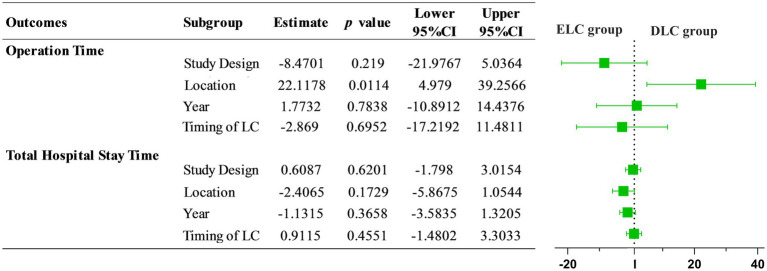
Meta regression of operation time and total hospital stay time between ELC and DLC. The results indicated that location factor was responsible for the source of heterogeneity with statistical significance (OR = 22.1178, 95%CI: 4.979–39.2566).

Furthermore, we analyzed the heterogeneity source using a Meta-regression analysis, revealing that the operation time and location accounted for the heterogeneity among studies (z = 2.5294, 95%CI: 4.9790–39.2566, *p* = 0.0114), consistent with the results of subgroup analysis (Figure 7).

## 4 Discussion

Since the first laparoscopic cholecystectomy was successfully performed in the late 1980s, minimally invasive surgery has been increasingly used to treat biliary tract diseases. Nowadays, LC has become the most common surgical approach for cholecystectomy. Due to the limitations of this new technology in the early days, laparoscopic cholecystectomy was not recommended for patients who suffered from acute cholecystitis due to the severe edema, the unclear anatomic structure of the Calot triangle, the uncontrollable bleeding around the gallbladder and the degree of surgeon’s experience (53–55). However, with the rapid development of laparoscopic technology and the refined understanding of intraoperative and postoperative complications of laparoscopic cholecystectomy, performing laparoscopic cholecystectomy for acute cholecystitis in the early period is no longer regarded as a contraindication (56, 57). Nevertheless, little is currently known about the optimal timing. Indeed, during the initial stages of acute cholecystitis, acute inflammatory reaction and edematous connective tissues impede the dissection of Calot’s triangle, contributing to reactive hyperemia and increase bleeding and bile duct injury during ELC. Accordingly, this may increase the operative time due to the severe inflammatory response in the early period of acute cholecystitis patients. Indeed, a longer operative time may increase the open surgery conversion rate and risk of biliary damage. On the other hand, patients that undergo DLC may benefit from a decreased conversion rate and risk of complications while prolonging hospitalization and increasing medical expenses (24, 58, 59).

Although several meta-analysis studies have compared ELC and DLC, they have some limitations. In this respect, Siddiqui et al. (60) and Gurusamy et al. (61) conducted studies based on a limited number of research and patients, suggesting significant bias in their studies. Complications of LC consist of intraoperative and postoperative complications. The most common intraoperative complications are bile duct injury, intraoperative bleeding, and conversion to open surgery. Bile duct injury is more serious and can be treated via *T*-tube placement. In cases of delayed diagnosis, ERCP may be performed after LC, but biliary stricture and recurrent biliary tract infection may occur. The most common postoperative complications are bile leak, wound site infection, and fluid collection around the gallbladder fossa. Bile leak may be the most serious among these, leading to acute peritonitis and septic shock. Current strategies to solve bile leakage include (1). Evaluation of the anatomical structure of the biliary tract; (2). For relatively small leaks, an indwelling abdominal tube should be placed for adequate drainage; (3). For relatively large leaks, a biliary stent may be inserted by ERCP (62, 63). Indeed, during the early stages of acute cholecystitis, gallbladder congestion, edema, brittle tissue bleeding and other factors may make laparoscopic dissection more challenging and constitute the main reason for conversion to laparotomy.

Our meta-analysis found that compared with ELC, DLC was associated with a high conversion rate and high postoperative complications with a fixed-effects model. However, the total hospitalization time in ELC was significantly shorter than in DLC when a random-effects model was utilized. In order to assess the heterogeneity in this meta-analysis, subgroup analysis and meta-regression were also employed, and both approaches indicated that regional factors accounted for the heterogeneity of this study. Subgroup analysis according to the operation time indicated that compared with Europe and America, a significantly longer operation time was observed for ELC patients in Asia, which suggested that acute inflammation and other factors during the early stage of acute cholecystitis were inclined to prolong the operation time in Asia (35, 36). During the subgroup analysis of hospitalization duration, we found that patients that underwent DLC in America correlated with shorter hospitalization than in Asia or Europe. The longest hospitalization duration was 7.10 ± 0.50 days (14) in America, which was shorter than in Asia (15.1 ± 11.1 days) (34) and Europe (20.5 ± 11.0 days) (26). This finding may account for the heterogeneity in our meta-analysis.

**Figure 8:**
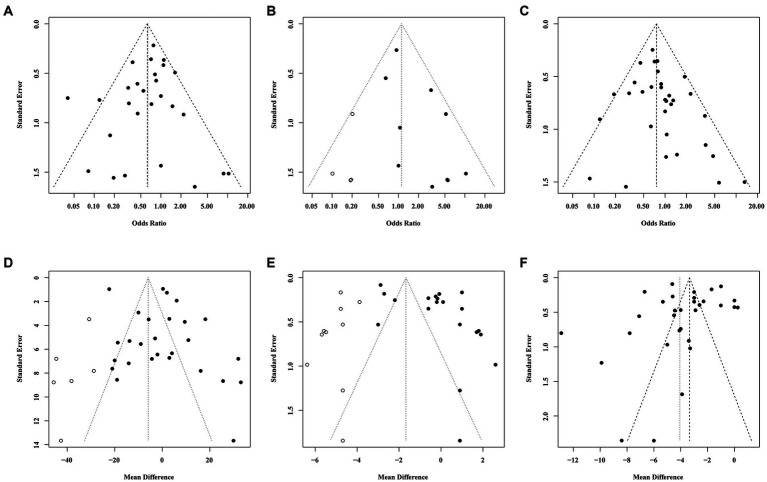
Funnel plots illustrated meta-analysis of primary and secondary outcomes. (**A–C**) represented the funnel plots of primary outcomes (**A**: Conversion rate; **B**: Intraoperative complications; **C**:Postoperative complications), the *x-axis* stood for odds ratio, while y-axis stood for standard error. (**D–F**) represented the funnel plots of secondary outcomes (**D**: Operation time; **E**: Postoperative hospital stay time; **F**: Total hospital stay time), the *x-axis* stood for mean difference while *y-axis* stood for standard error. Both funnel plots of **B,D,E** using trim-and-fill method, after filling studies indicated as hollow circle dots in the funnel plots, the funnel plot is basically symmetrical.

To investigate the stability and reliability of our findings, a sensitivity analysis was performed by removing one study each time. No study interfered with the results of this meta-analysis, substantiating the stability and reliability of our pooled estimates (Appendix 7). To analyze the possible presence of publication bias in this meta-analysis, we conducted an Egger’s test and generated funnel plots. Egger’s test showed that the funnel plots of intraoperative complications and postoperative hospital stay time were asymmetric. Next, we evaluated the impact of publication bias on the results by using the trim-and-fill method. It was found that with 4 added studies on intraoperative complications and 10 on postoperative hospitalization duration, there was no significant change in OR, WMD and their corresponding *p* values. Our funnel plot with filled-in data which was based on the trim-and-fill approach exhibited a symmetrical distribution (Figure 8).

Several limitations found in this meta-analysis should be acknowledged. First, about one-third of the studies were retrospective studies, and the blinding method was not mentioned, which impacted the quality of included studies. Besides, studies from different locations accounted for the heterogeneity in this meta-analysis. Selecting uniform RCTs might reduce the heterogeneity, but it would increase the risk of bias. Moreover, for publication bias on intraoperative complications and postoperative hospital stay time, although the funnel plot with filled-in data based on the trim-and-fill approach exhibited a symmetrical distribution, other factors such as study design, exceeding positive results and greater weight mean difference may account for publication bias. Accordingly, more large-scale, high-quality RCTs are required in the future.

## 5 Conclusion

This meta-analysis revealed that compared with DLC, ELC was associated with a lower conversion rate and incidence of postoperative complications and shorter hospitalization duration for acute cholecystitis. ELC brings significant advantages in terms of safety profile and cost-effectiveness. Nevertheless, despite our rigorous methodology, some limitations were still unavoidable. Large-scale and high-quality RCTs with long follow-ups are warranted to validate the findings of this meta-analysis.

## Data availability statement

The original contributions presented in the study are included in the article/Supplementary material, further inquiries can be directed to the corresponding authors.

## Author contributions

HW and KM: study concept and design. TJ and JH: acquisition of data. HW and BL: statistical analysis and manuscript writing. TC and YL: generation of statistical figures. HW, BL, TC, TJ, JH, YL, and KM: final approval of manuscript. All authors contributed to the article and approved the submitted version.
