# Supplementary material for: Comparison of the safety profile, conversion rate and hospitalization duration between early and delayed laparoscopic cholecystectomy for acute cholecystitis: a systematic review and meta-analysis
Source: Front Med (Lausanne). 2023 Dec 11;10:1185482. doi: 10.3389/fmed.2023.1185482 (PMC10750350; doi:10.3389/fmed.2023.1185482)
Supplement: Supplementary file 1 [file Table_1.DOCX]

**Comparison of the Safety profile, Conversion rate and Hospitalization duration between Early and Delayed Laparoscopic Cholecystectomy for Acute Cholecystitis: A Systematic Review and Meta-analysis**

**Appendix**


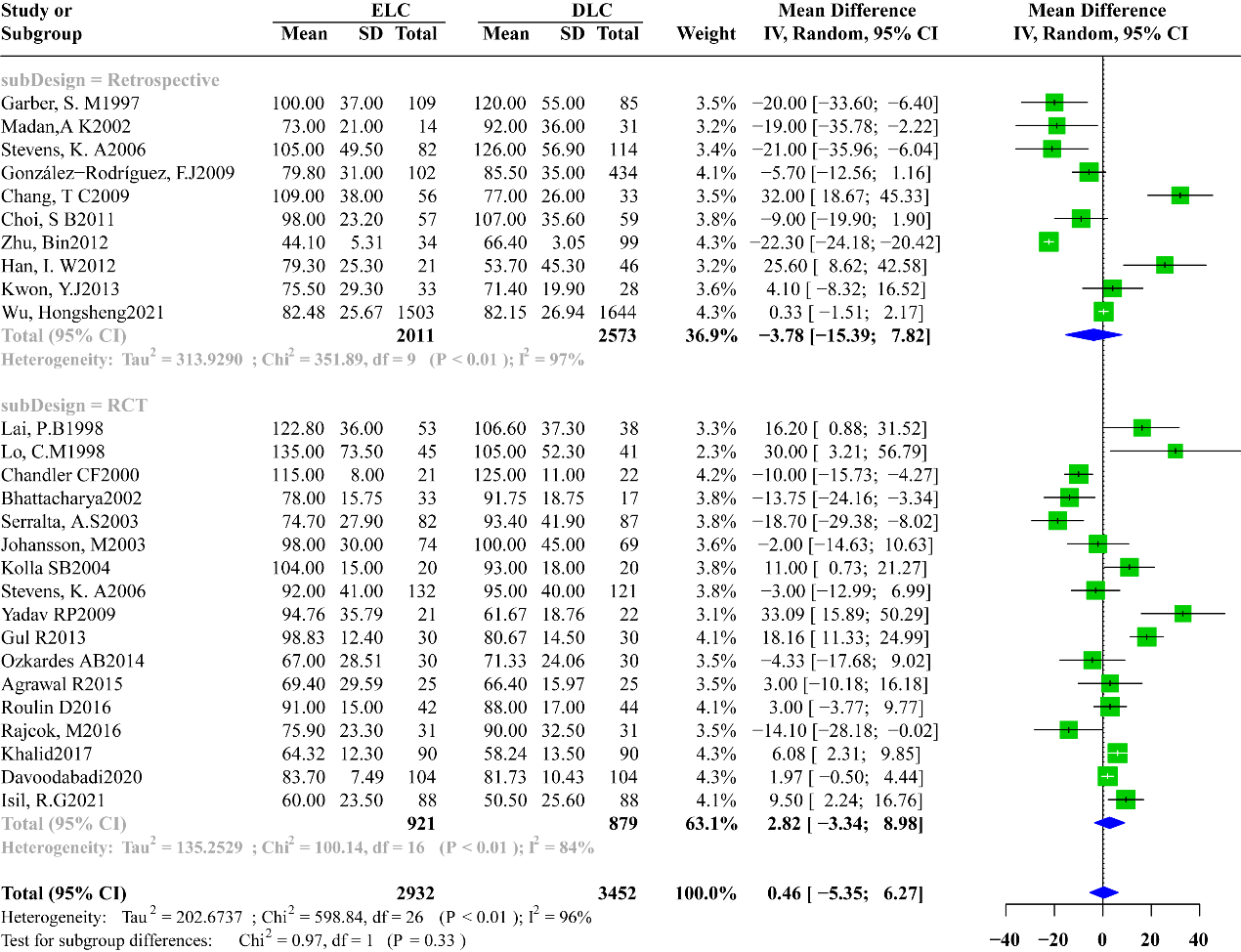


Appendix1 Subgroup analysis of study design for operation time between ELC and DLC.


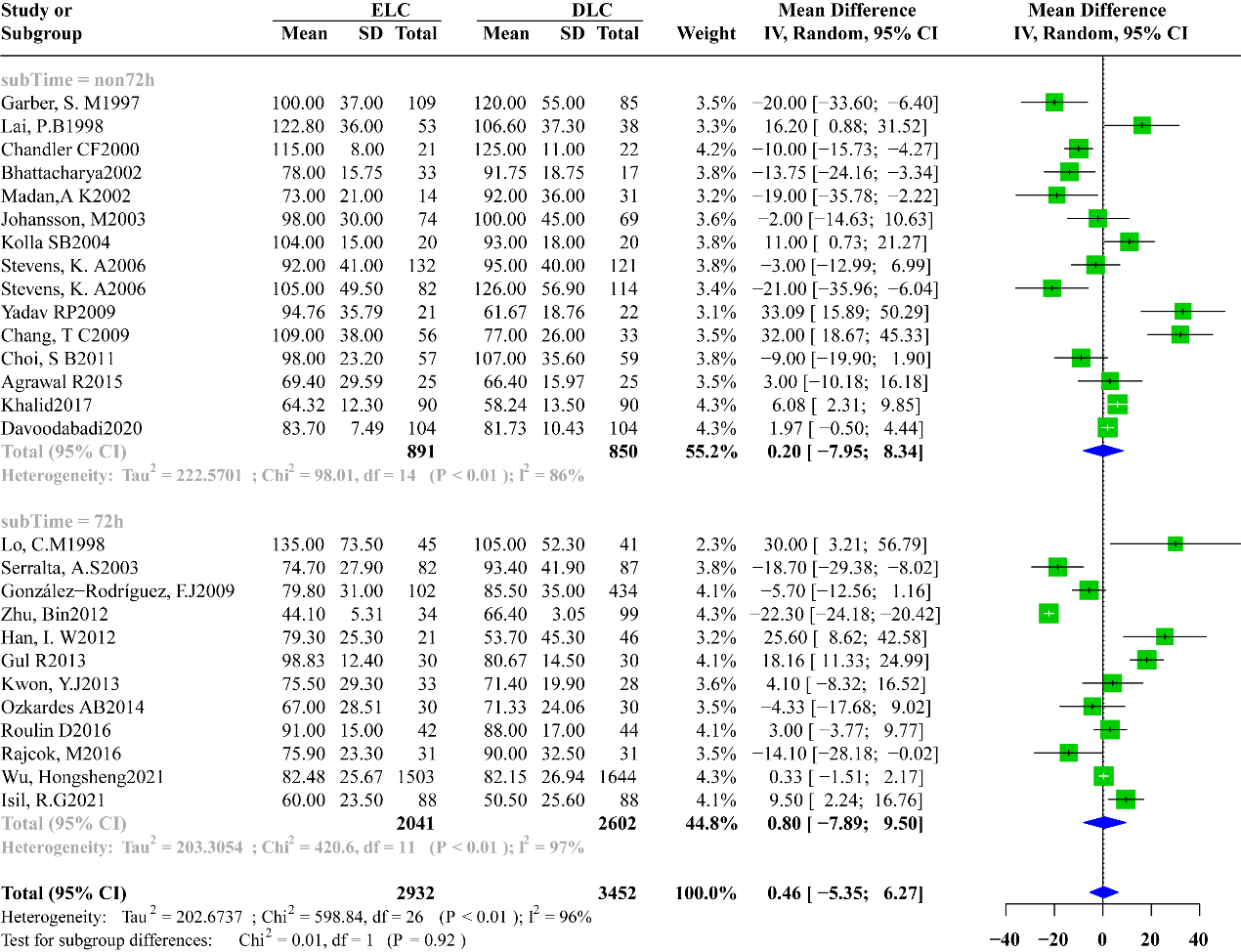


Appendix2 Subgroup analysis of definition of ELC(Timing of laparoscopic cholecystectomy after the onset of acute cholecystitis less than 72h or other definitions) for operation time between ELC and DLC.


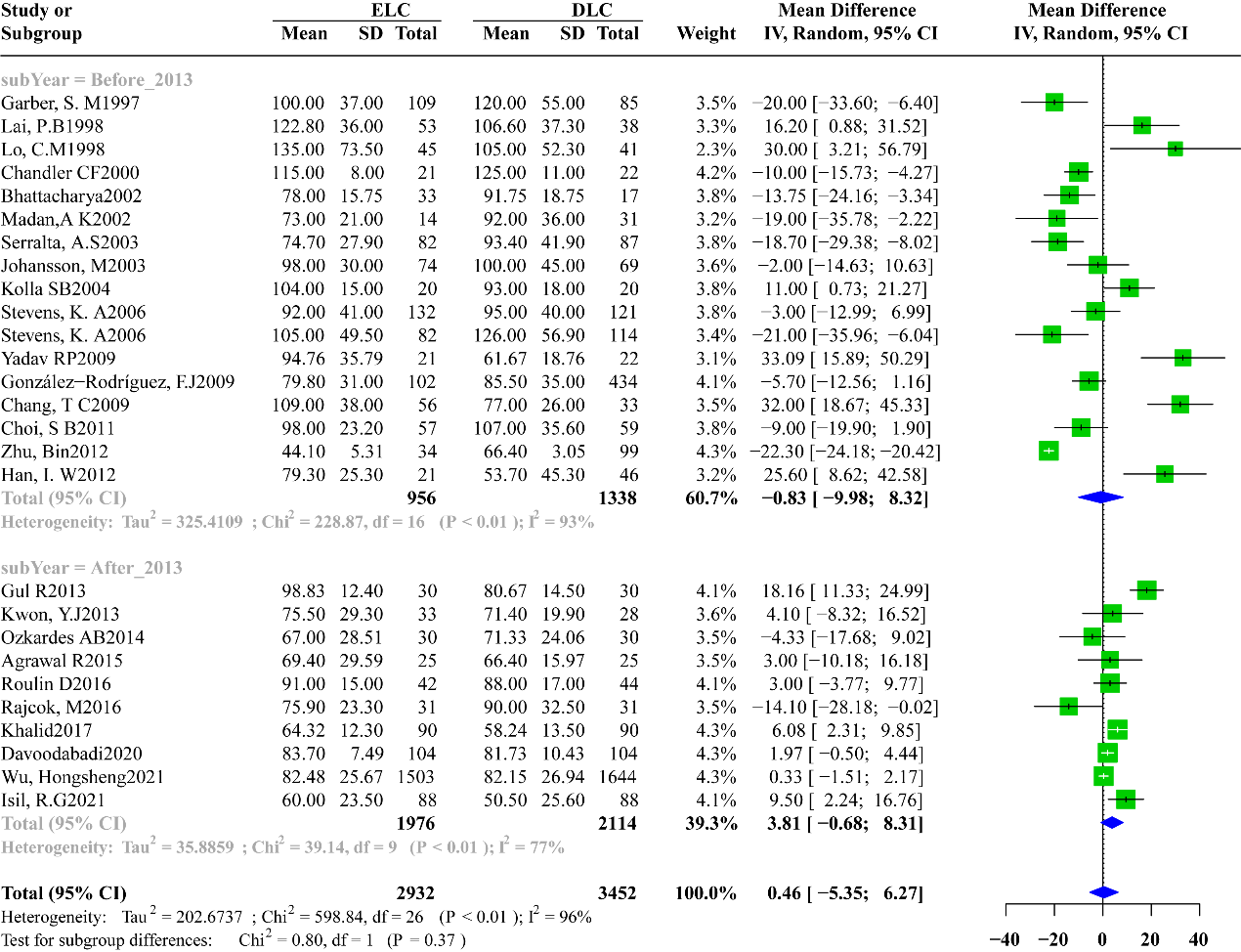


Appendix3 Subgroup analysis of year of study(Studies before 2013 or since and after 2013) for operation time between ELC and DLC.


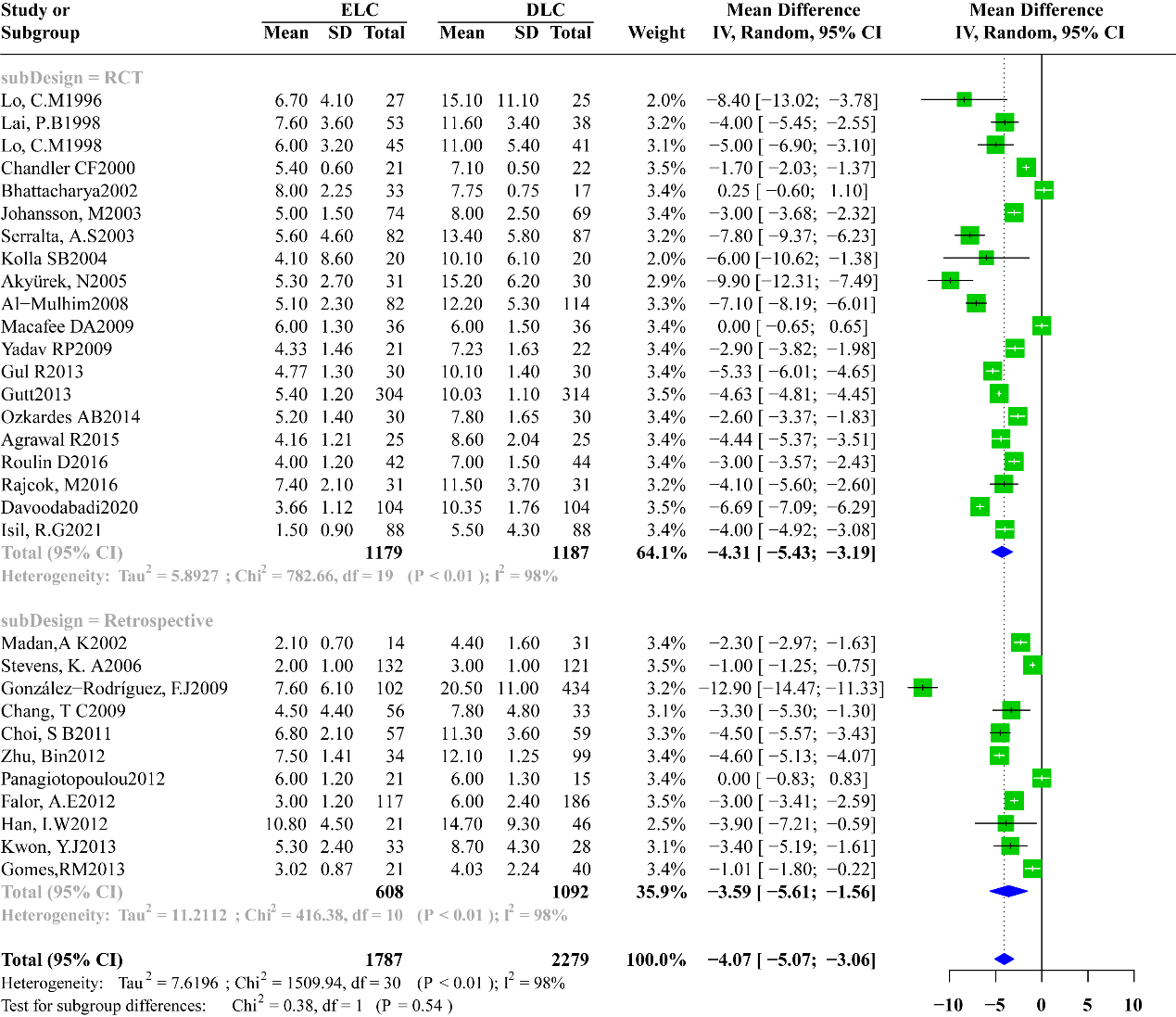


Appendix4 Subgroup analysis of study design for total hospital stay time between ELC and DLC.


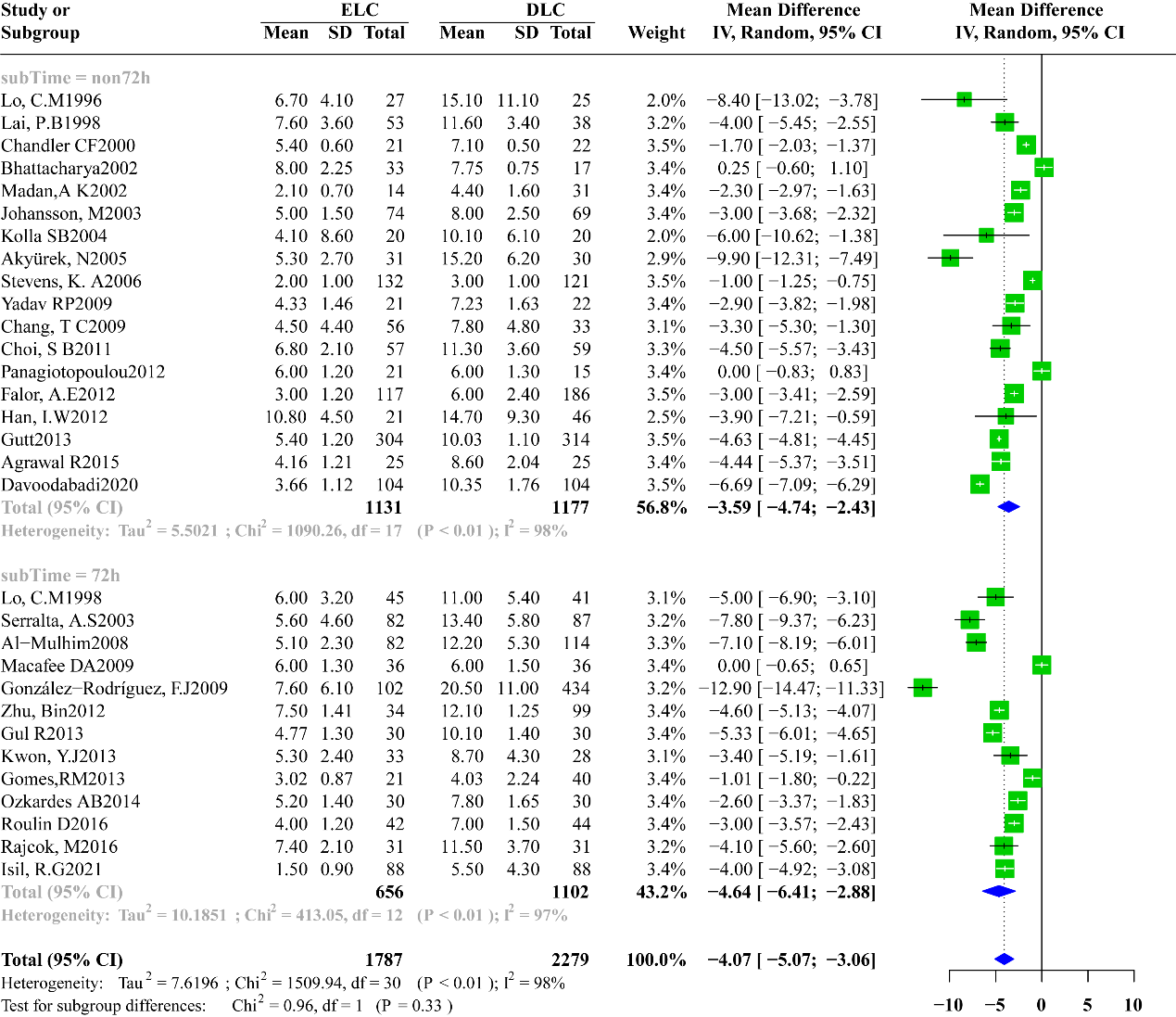


Appendix5 Subgroup analysis of definition of ELC(Timing of laparoscopic cholecystectomy after the onset of acute cholecystitis less than 72h or other definitions) for total hospital stay time between ELC and DLC.


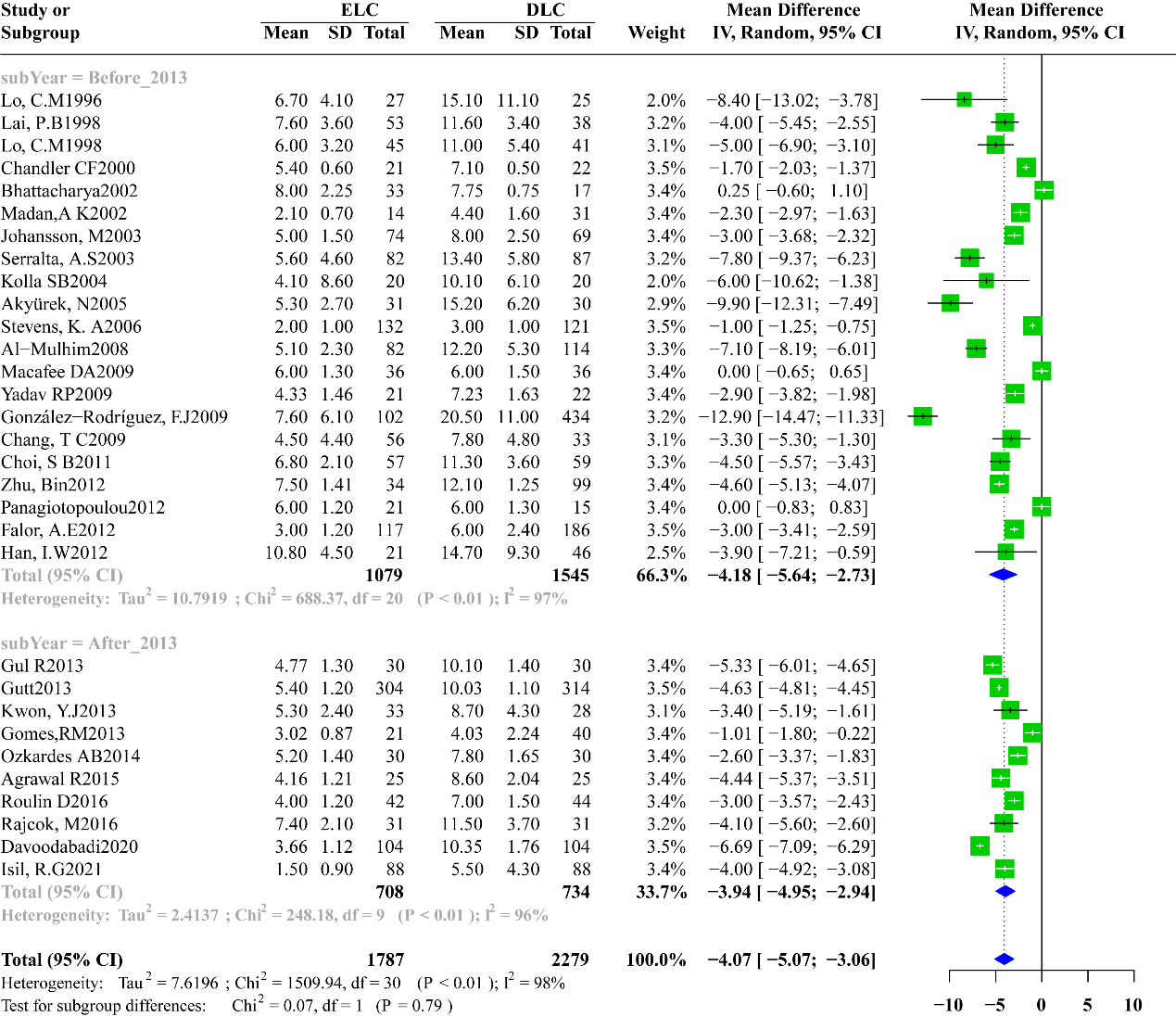


Appendix6 Subgroup analysis of year of study(Studies before 2013 or since and after 2013) for total hospital stay time between ELC and DLC.


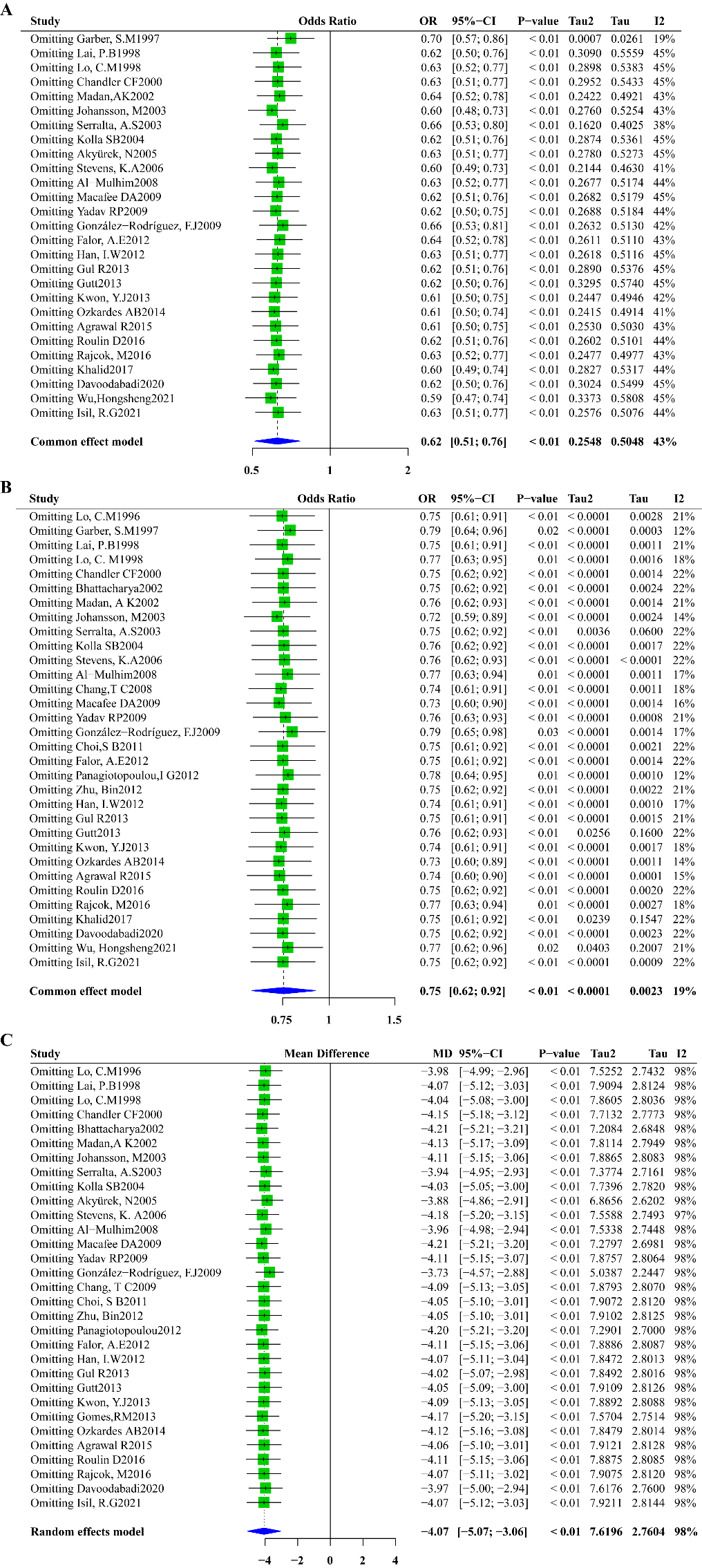


Appendix7 Sensitivity analysis of conversion rate(A), postoperative complications(B) and total hospital stay time(C).
